# Enhancing the Low-Temperature CO Oxidation over CuO-Based α-MnO_2_ Nanowire Catalysts

**DOI:** 10.3390/nano12122083

**Published:** 2022-06-16

**Authors:** Yan Cui, Huikang Song, Yiyu Shi, Pengxiang Ge, Mindong Chen, Leilei Xu

**Affiliations:** Collaborative Innovation Center of Atmospheric Environment and Equipment Technology, Jiangsu Key Laboratory of Atmospheric Environment Monitoring and Pollution Control, School of Environmental Science and Engineering, Nanjing University of Information Science & Technology, Nanjing 210044, China; cuiyan@nuist.edu.cn (Y.C.); nuistshk@163.com (H.S.); syyee@163.com (Y.S.); gepx@nuist.edu.cn (P.G.)

**Keywords:** CuO-based catalyst, α-MnO_2_ nanowire, low-temperature catalytic activity, CO oxidation

## Abstract

A series of CuO-based catalysts supported on the α-MnO_2_ nanowire were facilely synthesized and employed as the CO oxidation catalysts. The achieved catalysts were systematically characterized by XRD, SEM, EDS-mapping, XPS and H_2_-TPR. The catalytic performances toward CO oxidation had been carefully evaluated over these CuO-based catalysts. The effects of different loading methods, calcination temperatures and CuO loading on the low temperature catalytic activity of the catalyst were investigated and compared with the traditional commercial MnO_2_ catalyst with a block structure. It was found that the slenderness ratio of a CuO/α-MnO_2_ nanowire catalyst decreases with the increase in CuO loading capacity. The results showed that when CuO loading was 3 wt%, calcination temperature was 200 °C and the catalyst that was supported by the deposition precipitation method had the highest catalytic activity. Besides, the α-MnO_2_ nanowire-supported catalysts with excellent redox properties displayed much better catalytic performances than the commercial MnO_2_-supported catalyst. In conclusion, the CuO-based catalysts that are supported by α-MnO_2_ nanowires are considered as a series of promising CO oxidation catalysts.

## 1. Introduction

Carbon monoxide is generally considered to be a fuel, resulting from the incomplete combustion of fuel. The environmental pollution it causes has become a serious problem all over the world, which has a great impact on human health and living environment [1,2]. At present, CO degradation technologies mainly include adsorption, separation, biodegradation, combustion, plasma catalysis, photocatalysis, catalytic oxidation and so on [3]. Among them, catalytic oxidation has been widely used because of its advantages of high purification efficiency, low reaction temperature and low cost [4]. Therefore, the design and development of catalysts with advanced performance is the key to solve CO catalytic oxidation. It was found that noble metals (Au [5], Pt [6] and Rh [7]) as the active center supported on specific metal oxides (CeO_2_ [8], MnO_2_ [9], ZrO_2_ [10] and Fe_2_O_3_ [11]) with excellent oxygen storage capacity usually have high activity for CO catalytic oxidation. However, due to the high price and scarce resources of precious metals, their large-scale application is greatly limited. In the past few decades, various transition metal oxides (Co_3_O_4_, CuO, Fe_2_O_3_ and MnO_2_) have proved to show excellent catalytic activity in CO catalytic oxidation. Co_3_O_4_-based catalysts have been widely studied for their low temperature catalytic activity that is similar to that of noble metal catalysts. However, the rapid deactivation of Co_3_O_4_-based catalysts occurs under high humidity [12,13]. In contrast, CuO-based catalysts have poor low temperature activity but high humidity tolerance. Therefore, the preparation of CuO-based catalysts with excellent low temperature activity has always been a research focus and challenge in the field of CO catalytic oxidation [14].

In order to design and prepare highly efficient CuO-based catalysts, the effect of catalyst supports and preparation strategies have been extensively studied. Among many catalysts supports, MnO_2_ has been widely studied because of its low cost, environmental friendliness and high activity [15]. It is well known that the physicochemical properties of MnO_2_ with different morphologies are often different. The common one-dimensional structures of MnO_2_ include block structure, nanorods, nanotube and nanowires. Among these morphologies, MnO_2_ of a nanowire structure plays an important role because nanowire with a one-dimensional structure can be used as the basic assembly unit of two- or three-dimensional structural materials [16,17]. In addition, composites with novel structures and properties can be prepared easily. Compared with traditional MnO_2_ with a bulk structure, nanostructured MnO_2_ materials generally have better physicochemical properties, such as a higher specific surface area, lower density and adjustable chemical properties [18]. In addition, various studies have shown that MnO_2_ materials with nanowire morphology usually have a high surface area and strong metal-support interactions. Compared with other morphologies of MnO_2_ nanomaterials, MnO_2_ nanowire usually has higher catalytic activity [19,20]. Therefore, nanowire is the main form of MnO_2_ nanomaterials. MnO_2_ nanowires generally have more surface oxygen adsorption, stronger reducibility, higher specific surface area and lower Mn-O bond strength than MnO_2_ nanorods. Therefore, it has a better catalytic performance in the catalytic combustion of dimethyl ether and the catalytic oxidation of toluene. Saputra et al. [21] found that MnO_2_ nanowire exhibited higher activity than MnO_2_ nanorods and MnO_2_ nanofibers in the co-degradation of phenol by reactive oxygen species and hydroxyl groups. In addition, compared with nanorod and nanotube MnO_2_, MnO_2_ nanowires as supports have stronger interactions with Ag and exhibit higher toluene oxidation activity. Among the MnO_2_ nanomaterials (α-MnO_2_, β-MnO_2_), α-MnO_2_ nanowire also showed the best catalytic activity for CO, even after Co_3_O_4_ nanoparticles were decorated. Liang et al. [22] synthesized four MnO_2_ nanomaterials with different crystal types. They found that the order of activity of CO catalytic oxidation is α = δ > γ > β-MnO_2_ because the [2 × 2] and [1 × 1] tunnel structures of α-MnO_2_ can occupy more space to obtain more CO adsorption sites. On the other hand, the strong catalytic performance of α-MnO_2_ for CO oxidation is due to the prolonged length of the Mn-O bond that is caused by twisted [MnO_6_], which may be conducive to the fracture of Mn-O bond, thus promoting CO oxidation. Therefore, α-MnO_2_ reacts easily with CO, which further improves its catalytic performance.

In addition, various studies have been carried out to improve the CO catalytic oxidation activity of MnO_2_ nanowires. In order to achieve this goal, effective strategies, including element-doped surface engineering and combination with other active substances have been extensively developed [23]. Metal loading and doping are two commonly used modification methods for preparing catalysts. Both can significantly improve the catalytic activity of MnO_2_ nanowires [24,25]. The loading method can disperse the metal or metal oxide on the surface of the catalyst carrier highly uniformly, and the catalyst with higher activity can be obtained due to the formation of a strong metal-support interaction. The doping method is to replace the metal cations in the main metal oxides with different cations, which can greatly change the chemical bonding on the surface of the main metal oxides and improve their catalytic performance [26,27]. The active sites in the catalytic system may be oxygen atoms near the dopant or the dopant itself. Therefore, the activity of MnO_2_ nanowires can be further improved by dispersing more active components on their surfaces. Gao et al. [28] found that hydrothermal doping of Cu significantly improved the CO oxidation activity and water resistance of α-MnO_2_ nanowires. For γ-MnO_2_, Zn-doped MnO_2_ achieves a 90% CO conversion at 160 °C and shows the best CO oxidation activity in other elements that are doped with γ-MnO_2_. Li et al. [29] synthesized α-MnO_2_ with a porous surface structure by acid treatment. It has rich adsorption sites for O_2_, thus enhancing the catalytic oxidation activity of MnO_2_ to CO. In addition, combining Au, Ag and CuO with MnO_2_ nanomaterials can effectively improve the efficiency of CO catalytic oxidation. Xu et al. [30] also studied the effect of a Ag-supported catalyst on CO catalytic oxidation activity on α-MnO_2_ nanowires, and the T_90%_ of Ag/α-MnO_2_ could reach below 100 °C.

In this study, α-MnO_2_ material with a perfect nanowire structure was successfully prepared by a one-step hydrothermal method. The nanowires with excellent structural properties and thermal stability could be used as CuO-based catalyst supports. A series of CuO-based α-MnO_2_ nanowire catalysts were prepared by initial impregnation and deposition precipitation methods for CO catalytic oxidation reaction at a low temperature. The catalysts were characterized by X-ray powder diffraction (XRD), scanning electron microscopy (SEM), energy-dispersed spectroscopy (EDS) mapping, X-ray photoelectron spectroscopy (XPS), etc. The effects of different loading methods, calcination temperature, CuO loading and the mesoscopic structure of MnO_2_ on CO oxidation activity at low temperature were studied.

## 2. Materials and Methods

### 2.1. Synthesis of α-MnO_2_ Nanowire Support

The α-MnO_2_ nanowire support was synthesized by a hydrothermal method according to the scheme that was previously reported [31]. Specifically, 3 mmol of MnSO_4_·H_2_O was first dissolved in 40 mL of deionized water and stirred for 5 min until the MnSO_4_·H_2_O solution was clarified. Then, 2 mmol of KMnO_4_ was also dissolved in 40 mL of deionized water and stirred for 5 min. After that, the KMnO_4_ solution was gradually added to the MnSO_4_·H_2_O solution to obtain the brown suspension, and the stirring was continued for 30 min. The stirred brown suspension was transferred to a Teflon reactor and hydrothermal reaction at 160 °C for 12 h. The brown-black liquid that was obtained after the hydrothermal treatment was washed six times with ethanol. The solid that was obtained by centrifugation was placed in a vacuum drying oven at 100 °C for 12 h, and then the α-MnO_2_ nanowire support was obtained.

### 2.2. CuO-Based α-MnO_2_ Nanowire Catalyst Preparation

The CuO-based supported α-MnO_2_ nanowire catalysts containing *x* wt% CuO (*x* = m_CuO_/(m_CuO_ + m_support_) × 100%) were synthesized by a deposition precipitation method. To be specific, α-MnO_2_ nanowire was dispersed in Cu(NO_3_)_2_·3H_2_O solution, and then Na_2_CO_3_ (0.01 M) solution was added droplet by droplet to adjust the pH to 8~9. The mixed solution was fully stirred for 30 min and then stood for 1h. After filtration, it was washed with deionized water and dried for 12 h in an oven of 120 °C. Then, the CuO-based α-MnO_2_ nanowire catalysts with different CuO loading were obtained by calcination at 200 °C for 5 h and were denoted as *x*CuO/α-MnO_2_-200-DP (*x* = 1, 3, 5, 10, 20 and 30). Meanwhile, a series of catalysts defined as 3CuO/α-MnO_2_-*T*-DP (*T* = 120, 200, 300 and 400) were synthesized under the same preparation process at a different calcination temperature, where “*T*” refers to the calcination temperature of the catalyst. In order to clarify the influence of the loading mode on the performance of the catalyst, the CuO-based α-MnO_2_ nanowire catalyst by incipient impregnation method was denoted as 3CuO/α-MnO_2_-200-IMP.

In addition, the CuO that was supported on a commercial MnO_2_ catalyst (3CuO/C-MnO_2_-200-DP) indicated that the special morphology of α-MnO_2_ nanowire also promoted the catalytic activity of CO catalytic oxidation. The commercial MnO_2_ that was used in this study was the most common one in the market, and it was normal to have fewer impurities in the commercial MnO_2_.

### 2.3. Catalyst Characterizations

X-ray powder diffraction (XRD) patterns of all catalysts were performed on a Smart Lab/3 kW Intelligent multifunctional X-ray Diffractometer (Shimadzu, Kyoto, Japan) (Cu Kα radiation 40 kV/100 mA, the step of 5°/min, 2θ = 10–80°). Scanning electron microscopy (SEM) and energy-dispersed spectroscopy (EDS) mapping measurements of all catalysts were carried out on a scanning electron microscopy (FEI TECNAI G2 F20, Hillsboro, OR, USA). The sample was glued to the conductive adhesive, and gold spraying was performed for 45 s and 10 mA using Oxford Quorum SC7620 sputtering coater (Quorum, UK). The morphology of the sample was photographed with a ZEISS Gemini SEM 300 scanning electron microscope. The X-ray photoelectron spectroscopy (XPS) measurements were tested on an Escalab 250Xi (Thermo Fisher Scientific, Waltham, MA, USA) that was equipped with an Al Kα X-ray source to determine the elemental composition and chemical states of the elements. The powder of the sample was spread and coated on conductive tape on the sample holder. The binding energies were calibrated using the C 1s line at 284.5 eV as the reference.

H_2_ temperature-programmed reduction (H_2_-TPR) experiments were carried out in a self-made fixed-bed reactor (assemble). The consumption curve of H_2_ was recorded and analyzed by an online LC-D200 mass spectrometer (TILON, Seoul, Korea). A mixture of H_2_ (0.4 mL/min) and Ar (7.6 mL/min) was introduced into the reactor. After the H_2_ signal baseline (m/z = 2) was stabilized, a H_2_-TPR experiment was performed at a heating rate of 20 °C/min from room temperature to 800 °C.

### 2.4. Catalyst Evaluation

The catalytic activity of CO oxidation of the catalyst in this system had been tested in a vertical fixed-bed continuous flow reactor that was equipped with quartz tubes (I.D. = 10.00 mm). The temperature of the reaction should be the center temperature of the catalyst bed and it was detected and controlled by the thermocouple that was located in the center of the catalyst bed. The gas flows of the feed gases are controlled by the mass flow controllers (MFC, Brooks Instrument, Hatfield, UK) and used as feed 1 vol % CO, 20 vol. % O_2_ and balanced N_2_. The catalyst weighed 0.1 g and was injected with CO reaction gas with a total flow of 20 mL/min. The CO oxidation corresponding to the gas hourly space velocity (GHSV) was 12,000 mL/(g·h) gas, and the catalytic activity of CO oxidation over different catalysts was tested in the specified temperature range. Finally, a GC-680 gas chromatograph (Perkin Elmer, Waltham, MA, USA) with a thermal conductivity detector (TCD) was used for an on-line analysis of the outlet gas. The catalytic activity of the catalyst was reflected and expressed by CO conversion. The conversion rate of CO was calculated based on the formula below:

(1) $$C_{CO}=\frac{F_{CO,Inlet}-F_{CO,Outlet}}{F_{CO,Intlet}}\times 100\%$$

*F_CO,Inlet_* represented the flow rate of CO species into; *F_CO,outlet_* represented the flow rate of CO species out of the reactor.

## 3. Results and Discussion

### 3.1. Characterizations of the Catalysts

#### 3.1.1. XRD Analysis

**Figure 1:**
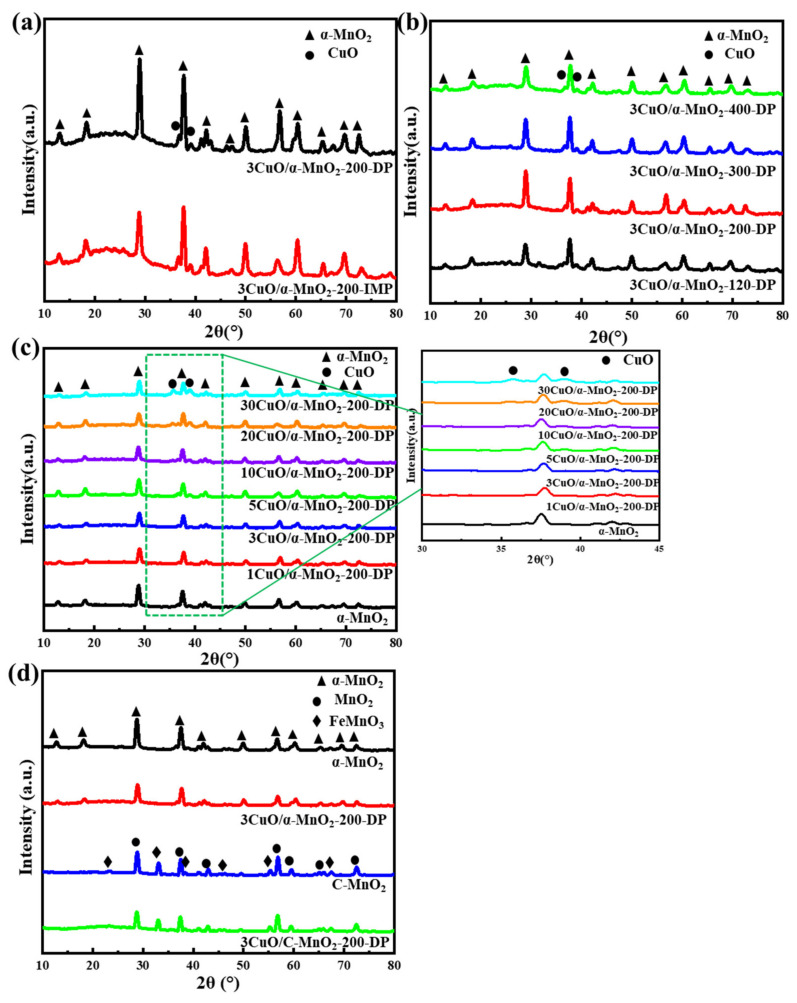
XRD patterns of (**a**) 3CuO/α-MnO_2_-200-DP and 3CuO/α-MnO_2_-200-IMP catalysts under different loading methods; (**b**) 3CuO/α-MnO_2_-*T*-DP (*T* = 120, 200, 300, 400) catalysts with different calcination temperatures; (**c**) *x*CuO/α-MnO_2_-200-DP (*x* = 0, 1, 3, 5, 10, 20, 30) catalysts with different CuO loading; (**d**) α-MnO_2_ nanowire, commercial MnO_2_ (C-MnO_2_) and corresponding catalysts.

In order to study the crystal phase structure of the supports and catalysts, a series of materials were analyzed by XRD. In Figure 1, the diffraction peaks of α-MnO_2_ that were located at 2θ = 12.78°, 18.11°, 25.71°, 37.52°, 41.97°, 49.86°, 56.37°, 65.11° and 69.71° could be ascribed to the α-MnO_2_ phase (PDF#44-0141) [30]. Specific, obvious diffraction peaks correspond to the (1 1 0), (2 0 0), (2 2 0), (2 1 1), (3 0 1), (4 1 1), (6 0 0), (5 2 1), (0 0 2) and (5 4 1) crystal planes of the MnO_2_ structure, respectively. Figure 1a shows the XRD patterns of 3CuO/α-MnO_2_-200-DP and 3CuO/α-MnO_2_-200-IMP under different loading methods. As can be seen from the figure, the MnO_2_ diffraction peak intensity of the 3CuO/α-MnO_2_-200-DP catalyst that was prepared by the deposition precipitation method (DP) loaded with CuO was significantly stronger than 3CuO/α-MnO_2_-200-IMP that was prepared by the initial impregnation method (IMP). The catalytic activity test showed that the CO catalytic oxidation activity of 3CuO/α-MnO_2_-200-DP was significantly better than that of 3CuO/α-MnO_2_-200-IMP.The test also proved that the (2 2 0) and (6 0 0) crystal planes of α-MnO_2_ may play a dominant role in CO catalytic oxidation. At the same time, compared with the 3CuO/α-MnO_2_-200-IMP, the characteristic peak of CuO in the 3CuO/α-MnO_2_-200-DP catalyst was much lower. The results showed that the CuO dispersion on the surface of the 3CuO/α-MnO_2_-200-DP catalyst was significantly higher than that of 3CuO/α-MnO_2_-200-IMP.

In general, calcination at higher temperatures was always accompanied by an increase in mean particle size and a decrease in specific surface area due to pore clogging. In addition to reducing the surface area, higher calcination temperatures reduced the active ingredients, ultimately leading to a reduction in the active interface sites. On the other hand, the CuO/α-MnO_2_ catalyst generated CuMn_2_O_4_ at higher temperature and deactivates [32]. Figure 1b shows the XRD patterns of 3CuO/α-MnO_2_-*T*-DP at different calcination temperatures. When the calcination temperature raised from 120 °C to 300 °C, the intensity of the XRD diffraction peak increased and the diffraction peak was the strongest at 300 °C. This ought to be attributed to the collapse of the nanowire framework at the calcination temperature as high as 300 °C, which made the dispersion of CuO worse. However, when the calcination temperature raised to 400 °C, the peak intensity decreased, which was caused by the formation of CuMn_2_O_4_. Moreover, due to the low content of CuMn_2_O_4_, its characteristic diffraction peak cannot be displayed in the XRD pattern. 

The XRD patterns of the pure α-MnO_2_ nanowire support and *x*CuO/α-MnO_2_-200-DP catalysts with different CuO loading are shown in Figure 1c. As can be seen from the figure, almost all the *x*CuO/α-MnO_2_-200-DP nanowire catalysts showed wide and clear XRD peaks, indicating that all the catalysts displayed good crystallinity. As the CuO loading increased, two diffraction peaks were detected at the 2θ = 35.5° and 38.8°, which were the diffraction peaks of CuO (PDF#05-0661) [33]. On the other hand, with the increase in CuO content, the intensity of the CuO diffraction peak also increased, indicating that the grain size of CuO increased. At the same time, the characteristic peak intensity of the (2 2 0) and (2 1 1) crystal planes of α-MnO_2_ decreased obviously.

Figure 1d shows the XRD patterns of pure α-MnO_2_ nanowire, commercial MnO_2_ and corresponding catalysts. As can be seen from the figure, the diffraction peak of C-MnO_2_ was the same as that of MnO_2_ (PDF#72-1984). In addition, the diffraction peak of impurity FeMnO_3_ (PDF#75-0894) was observed in the C-MnO_2_ diffraction peak by comparison [34]. The characteristic peak of CuO cannot be clearly seen in the figure, due to the low loading of CuO. On the other hand, it indicated the high dispersion of CuO on the catalyst’s surface.

#### 3.1.2. SEM Observation

**Figure 2:**
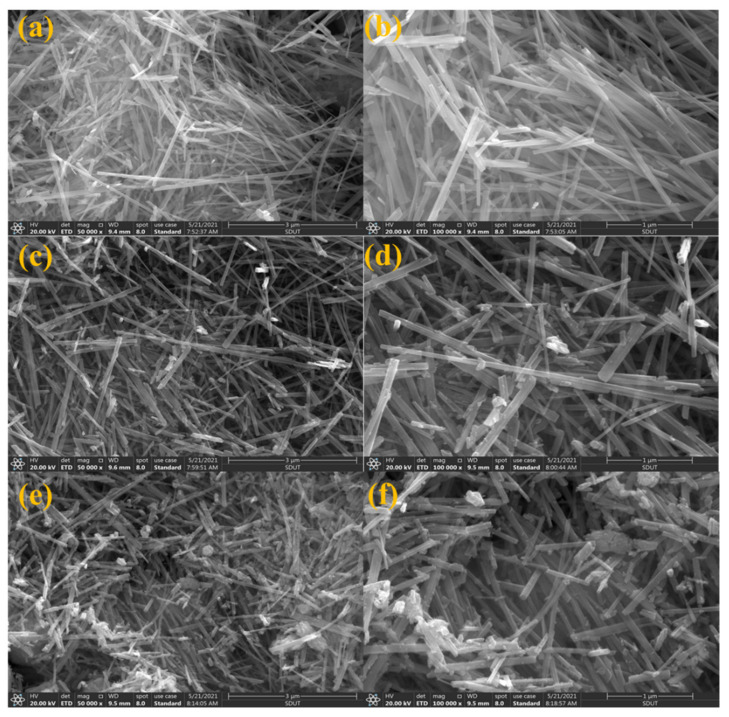
SEM images of α-MnO_2_ nanowire support (**a**,**b**), 5CuO/α-MnO_2_-200-DP (**c**,**d**) and 30CuO/α-MnO_2_-200-DP (**e**,**f**) catalysts.

SEM images of the α-MnO_2_ nanowire support and *x*CuO/α-MnO_2_-200-DP catalysts were analyzed, as shown in Figure 2. The 5CuO/α-MnO_2_-200-DP and 30CuO/α-MnO_2_-200-DP with different CuO loadings were selected as representative catalysts. Figure 2a,b shows the morphology of pure α-MnO_2_ nanowire. It was found that the nanowire had a uniform morphology, smooth surface, and large aspect ratio (length: 5–15 μm, width: 100–200 nm). Figure 2c,e, respectively, show the 5CuO/α-MnO_2_-200-DP and 30CuO/α-MnO_2_-200-DP nanowires’ catalysts’ structure. When the CuO loading increased from 0% to 5%, α-MnO_2_ nanowire support retained its morphology, while the 30% CuO loading catalyst showed irregular nanowire. At the same time, the aspect ratio of α-MnO_2_ nanowires decreases with the increase in CuO loading. The CuO loading was observed on the surface of the 5CuO/α-MnO_2_-200-DP and 30CuO/α-MnO_2_-200-DP catalysts.

**Figure 3:**
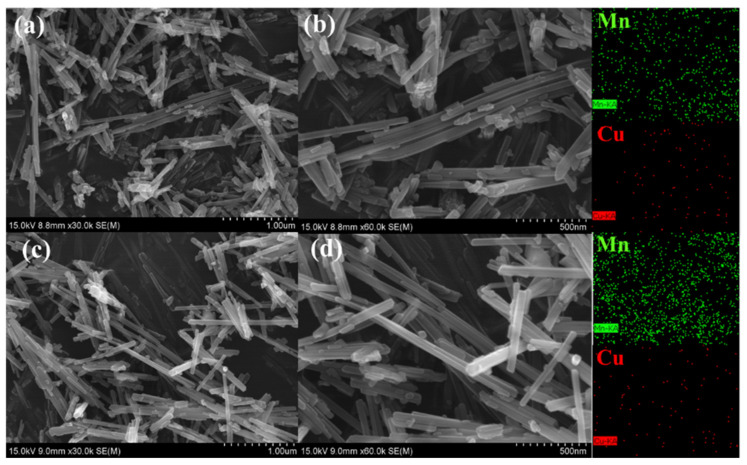
STEM and EDS element mapping images showing the spatial distribution of Mn and Cu elements: (**a**,**b**) 3CuO/α-MnO_2_-200-DP, (**c**,**d**) 3CuO/α-MnO_2_-200-IMP.

The spatial dispersion of Mn and Cu elements in the nanowire structure could be characterized and analyzed by scanning transmission electron microscopy (STEM) and energy dispersive spectroscopy mapping (EDS-mapping) of the 3CuO/α-MnO_2_-200-DP and 3CuO/α-MnO_2_-200-IMP catalysts. It could be seen from Figure 3 that the supported metal Cu element was uniformly distributed on the surface of the catalysts. In addition, the dispersion of Cu element in the 3CuO/α-MnO_2_-200-DP catalyst was significantly higher than that of the 3CuO/α-MnO_2_-200-IMP catalyst, indicating that the catalyst that was prepared by the precipitation deposition method could better distribute the Cu element on the catalyst’s surface.

#### 3.1.3. XPS Analysis

**Figure 4:**
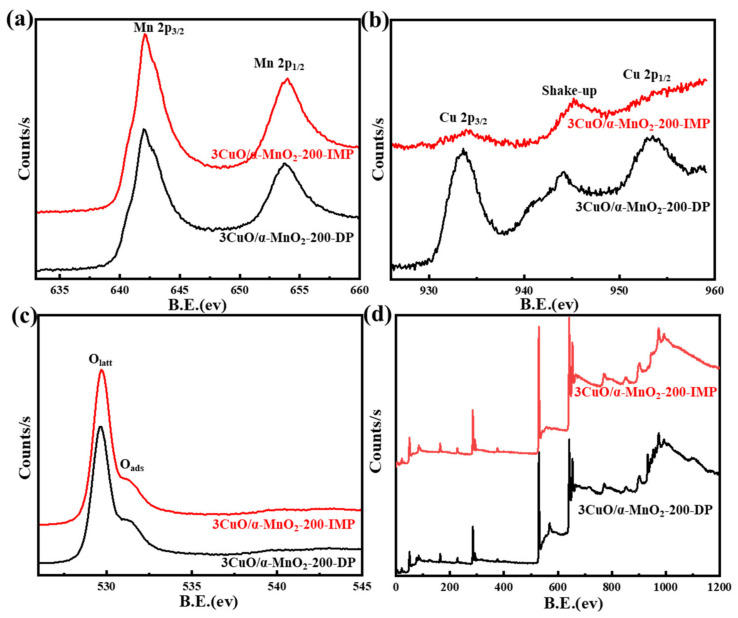
XPS spectra of Mn 2p (**a**), Cu 2p (**b**)**,** O 1s (**c**) and survey spectrum (**d**) for 3CuO/α-MnO_2_-200-DP and 3CuO/α-MnO_2_-200-IMP catalysts under different loading methods.

**Figure 5:**
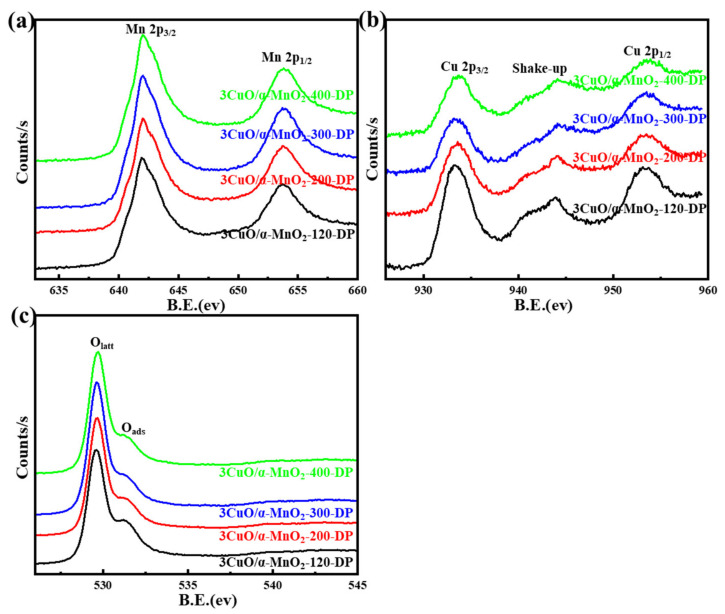
XPS spectra of Mn 2p (**a**), Cu 2p (**b**) and O 1s (**c**) for 3CuO/α-MnO_2_-*T*-IMP catalysts with different calcination temperatures.

**Figure 6:**
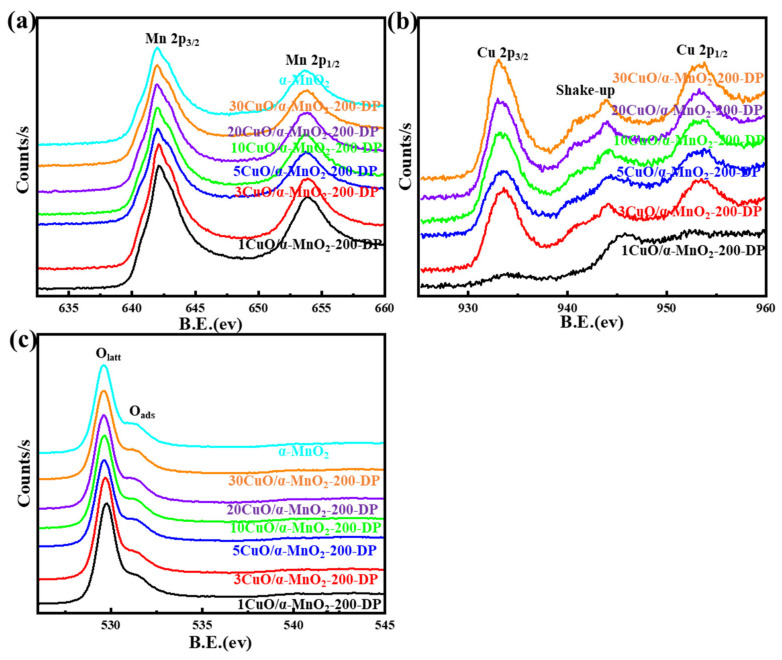
XPS spectra of Mn 2p (**a**), Cu 2p (**b**) and O 1s (**c**) for *x*CuO/α-MnO_2_-200-DP catalysts with different CuO loading.

**Figure 7:**
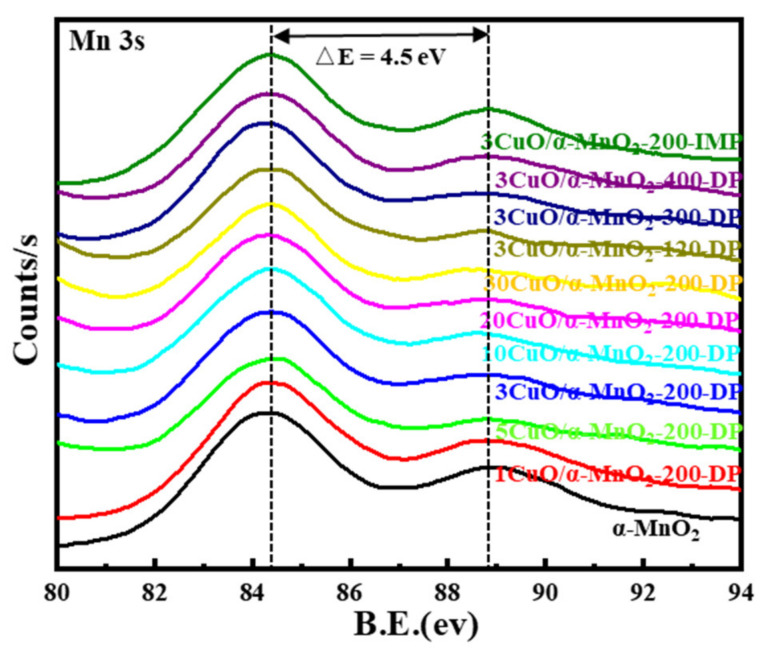
XPS spectra of Mn 3s for catalysts.

An XPS analysis of a series of prepared catalysts provided further evidence of surface chemical coordination, valence and composition states. Their XPS curves were shown in Figure 4, Figure 5, Figure 6 and Figure 7. It can be seen from Figure 7 that the XPS spectrum of Mn 3s had a double peak structure, and the double peak spacing of all catalysts was 4.5 eV. Therefore, it could be concluded that the Mn species in all catalysts existed in the form of MnO_2_. It can be seen from Figure 4a,c that the XPS distribution of Mn 2p and O 1s catalysts under different loading modes was almost the same, because the addition of a small amount of CuO did not affect the overall element concentration ratio of Mn and O. It can be observed from Figure 4b that these catalysts have two main peaks at 933.3 eV and 953.03 eV, which may be attributed to Cu 2p_3/2_ and Cu 2p_1/2_, respectively. In addition, it was noteworthy that the Cu 2p_3/2_ peak was almost accompanied by an oscillating satellite peak in the range of 940.38–943.28 eV. This was accompanied by three satellite peaks with Cu 2p_3/2_ peaks at 940.4 eV (I), 941.8 eV (II), and 943.4 eV (III). It was well known that the satellite peak was caused by the transfer of electrons from the ligand orbit to the 3d orbit of Cu, which confirmed the existence of Cu^2+^ in the divalent form of the 3d^9^ structure, rather than the species level of Cu^+^ or Cu^0^ with d-filled energy [35,36]. Meanwhile, the XPS spectrum of Cu 2p varies greatly under different loading modes. The peak intensity of the Cu 2p spectrum of the 3CuO/α-MnO_2_-200-IMP catalyst was significantly lower than that of the 3CuO/α-MnO_2_-200-DP catalyst. The peak intensity of the Cu 2p spectrum of the 3CuO/α-MnO_2_-200-IMP catalyst was significantly lower than that of the 3CuO/α-MnO_2_-200-DP catalyst. The main reason for this was the different CuO dispersion over these catalysts. Specifically, the CuO dispersion of the 3CuO/α-MnO_2_-200-IMP catalyst was poorer than the 3CuO/α-MnO_2_-200-DP catalyst. According to the XPS survey spectra results (Table 1), the surface concentration of the Cu element over the 3CuO/α-MnO_2_-200-DP catalyst was 2.9%, which was similar to the content of the Cu element that was added. However, the surface concentration (1.7%) of the Cu element in the 3CuO/α-MnO_2_-200-IMP catalyst was much lower than the theoretical value. These results indicated that the deposition-precipitation method could better disperse the CuO than the initial impregnation method over the α-MnO_2_ nanowire support.

The XPS spectra of Mn 2p, O 1s and Cu 2p at different calcination temperatures of the 3CuO/α-MnO_2_-*T*-DP catalyst are shown in Figure 5. As can be seen from Figure 5a, these catalysts had two main peaks at 654.0 eV and 642.1 eV, which belong to Mn 2p_1/2_ and Mn 2p_3/2_ spin orbits, respectively. It should be noted that these two peaks were characteristic signals of Mn (IV). All these indicated the occurrence of interfacial reactions and the formation of MnO_2_. To show the redox characteristics of the prepared catalyst, the surface oxidation state of copper was also studied [37]. As can be seen from Figure 5b, the XPS peaks that are centered on 954.0 eV and 933.0 eV belong to Cu 2p_1/2_ and Cu 2p_3/2_, respectively. The binding energy of Cu 2p increased with the increase in calcination temperature. Meanwhile, with the increase in calcination temperature, the peak intensity corresponding to Cu 2p decreased, which may be caused by the decrease in Cu species’ concentration on the catalyst surface, caused by the increase in calcination temperature. To further investigate the properties of various oxygen substances on the 3CuO/α-MnO_2_-*T*-DP catalyst, the XPS spectrum of O 1s of 3CuO/α-MnO_2_-*T*-DP are shown in Figure 5c. All the catalysts show two peaks of different oxygen species. Specifically, the peaks of 529.9 eV and 531.2 eV can be attributed to the lattice oxygen (O_latt_) and surface-adsorbed oxygen (O_ads_) of CuO_x_ and α-MnO_2_, respectively. Combined with the data after peak fitting in Table 2, with the increase in calcination temperature, the acromial area ratio also decreased correspondingly. The oxygen vacancy concentration of 3CuO/α-MnO_2_-200-DP was the highest. According to previous reports [38], the formation of oxygen anion radicals was due to the increased adsorption of environmental oxygen by surface oxygen vacancies, which will further improve the performance of catalysts.

Figure 6 shows the XPS spectra of Mn 2p, O 1s and Cu 2p in *x*CuO/α-MnO_2_-200-DP catalysts with different CuO loadings. As can be seen from Figure 6a, these catalysts had two main peaks at 652.7 eV and 642.0 eV, which were Mn 2p_1/2_ and Mn 2p_3/2_, respectively. This indicated that Mn existed in the form of Mn^4+^ in the *x*CuO/α-MnO_2_-200-DP catalyst. In order to show the redox characteristics of the prepared catalyst, the surface oxidation state of copper was also studied [39]. As can be seen from Figure 6b, these catalysts had two main peaks at 953.0 eV and 933.0 eV, namely Cu 2p_1/2_ and Cu 2p_3/2_. It was noteworthy that the peak intensity of the Cu 2p spectrum increased significantly with the increase in copper content, and the corresponding peak fitting results also showed that the proportion of Cu element increased. In order to clarify the properties of various oxygen-containing substances on the *x*CuO/α-MnO_2_-200-DP catalyst, the XPS spectra of O 1s of all the *x*CuO/α-MnO_2_-200-DP catalysts are shown in Figure 6c. According to the binding energy of surface elements, the binding energy of the *x*CuO/α-MnO_2_-*T*-DP catalyst in Table 3 decreased with the increase in CuO loading. Combined with the data after peak fitting in Table 2, 3CuO/α-MnO_2_-200-DP had the highest acromion area ratio of O 1s. Metal-support interactions between well-dispersed CuO and MnO_2_ support produce more surface oxygen and defects. These oxygen and defects predominated when the catalysts exhibited strong CO oxidation properties, and high oxygen vacancy provided a higher catalyst performance for CO catalytic oxidation. In conclusion, different CuO loadings and calcination temperatures together affect the formation of oxygen vacancy on the surface of the catalyst, and further affect the catalytic oxidation performance of CO.

#### 3.1.4. H_2_-TPR Analysis

In order to study the interaction between catalyst support and CuO, a H_2_-TPR analysis was systematically carried out on the catalysts of commercial MnO_2_ and α-MnO_2_ nanowire that were loaded with CuO under different CuO loadings, different calcination temperatures and different loading methods. The typical H_2_-TPR curve is shown in Figure 7.

**Figure 8:**
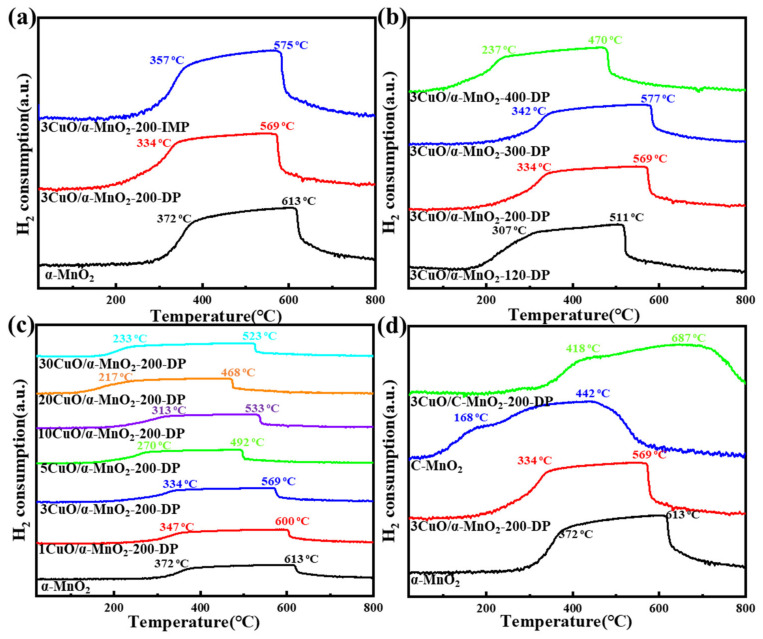
H_2_-TPR profiles of (**a**) α-MnO_2_, 3CuO/α-MnO_2_-200-DP and 3CuO/α-MnO_2_-200-IMP catalysts under different loading methods; (**b**) 3CuO/α-MnO_2_-*T*-DP (*T* = 120, 200, 300, 400) catalysts with different calcination temperatures; (**c**) *x*CuO/α-MnO_2_-200-DP (*x* = 0, 1, 3, 5, 10, 20, 30) catalysts with different CuO loading; (**d**) α-MnO_2_ nanowire, commercial MnO_2_ (C-MnO_2_) and corresponding catalysts.

Figure 8a shows the H_2_-TPR curves of α-MnO_2_ nanowire catalyst under different loading modes. With the loading of CuO, the reduction peak of the catalyst moved towards a low temperature, and the reduction in catalyst by precipitation deposition was improved more obviously. This meant that the interaction between CuO and α-MnO_2_ nanowire was stronger. Figure 8b shows the H_2_-TPR curves of the 3CuO/α-MnO_2_-*T*-DP nanowire catalysts that were calcined at 120 °C, 200 °C, 300 °C and 400 °C. With the increase in calcination temperature, the relative position between the two reduction peaks of the catalyst basically remained unchanged, and the H_2_-TPR curve of the catalysts that were calcined at 200 °C and 300 °C moved to a higher temperature. The increase in CuO composition at this temperature required a higher temperature to completely reduce the catalyst. This indicated that the excellent reduction performance could be reduced by high loading. Figure 8c shows the H_2_-TPR curve of α-MnO_2_ nanowire with two main peaks, the maximum values of which were concentrated at 372 °C and 613 °C, respectively. The first peak at 372 °C was attributed to the reduction in MnO_2_ to Mn_3_O_4_, while the second peak at 613 °C was attributed to the reduction in Mn_3_O_4_ to MnO [40]. However, after loading different amounts of CuO, all the *x*CuO/α-MnO_2_-200-DP catalysts showed a two-stage reduction peak like that of pure α-MnO_2_ nanowires, and there was no characteristic reduction peak of CuO species. Meanwhile, the addition of CuO to α-MnO_2_ nanowire changed the reduction behavior of α-MnO_2_ nanowire. With the increase in CuO loading, the continuous reduction peaks of MnO_2_ to Mn_3_O_4_ and Mn_3_O_4_ to MnO also shift to lower temperatures. This finding clearly indicated that the CuO had a significant effect on the reduction performance of the *x*CuO/α-MnO_2_-200-DP catalyst. Figure 8d shows the H_2_-TPR curves of commercial MnO_2_ and α-MnO_2_ nanowire catalysts before and after CuO loading. Unlike α-MnO_2_ nanowire, the reduction peak of the commercial MnO_2_ catalyst that was loaded with CuO shifts to a high temperature. This meant that the reduction performance of commercial MnO_2_ that was loaded with CuO was significantly reduced, indicating that the interaction between CuO and commercial MnO_2_ was weak. The structural advantages of α-MnO_2_ nanowires and their ability to interact with oxides were shown from the side view.

### 3.2. Catalytic Performance for CO Oxidation

#### 3.2.1. Catalytic Activity

**Figure 9:**
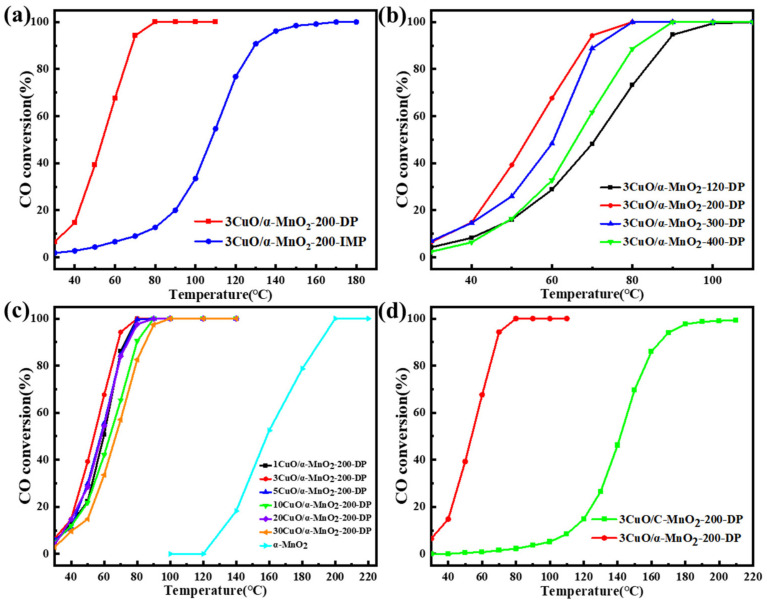
(**a**) 3CuO/α-MnO_2_-200-DP and 3CuO/α-MnO_2_-200-IMP catalysts under different loading modes; (**b**) 3CuO/α-MnO_2_-*T*-DP (*T* = 120, 200, 300, 400) catalysts; (**c**) *x*CuO/α-MnO_2_-200 (*x* = 0, 1, 3, 5, 10, 20, 30) catalyst; and (**d**) CO conversion of pure α-MnO_2_ nanowires and commercial MnO_2_ catalysts supported by CuO by precipitation deposition at different reaction temperatures.

In order to study the influence of the loading mode on the catalytic activity, the catalytic oxidation reaction of 3CuO/α-MnO_2_-200-DP (deposition precipitation method) and the 3CuO/α-MnO_2_-200-IMP (initial impregnation method) catalyst CO was studied. The results are shown in Figure 9a. As can be seen in the figure, the 100% CO conversion temperature of the 3CuO/α-MnO_2_-200-DP catalyst was 80 °C. The catalytic activity of the 3CuO/α-MnO_2_-200-IMP catalyst was much lower than that of the 3CuO/α-MnO_2_-200-DP catalyst, and the complete transformation of CO can be realized until 170 °C. This shows that the sedimentation method has obvious advantages over the traditional initial impregnation method.

To study the influence of calcination temperature on the catalytic activity of the 3CuO/α-MnO_2_-*T*-DP catalyst, the CO catalytic oxidation reaction was studied at calcination temperatures of 200 °C, 300 °C and 400 °C, and catalyst samples dried only at 120 °C without calcination, as shown in Figure 9b. As can be seen from the figure, the catalytic activity of 3CuO/α-MnO_2_-*T*-DP catalyst decreases as the calcination temperature increases from 200 °C to 400 °C. The catalytic activity of catalysts that have been calcined at 120 °C, was lower than that of the catalyst samples that have been calcined at 200 °C to 400 °C. This may be because Cu(NO_3_)_2_·3H_2_O did not decompose completely due to the low temperature of catalyst samples that were calcined above 200 °C during the loading process. The calcination temperature of the catalysts had great influence on the dispersion, structure and metal–surface interaction strength of the catalysts. It can be seen from the XRD pattern that the CuO diffraction peak intensity of 3CuO/α-MnO_2_-*T*-DP catalyst was different to some extent. The CuO diffraction peak of 3CuO/α-MnO_2_-300-DP was the strongest, indicating that the CuO species were poorly dispersed. Therefore, the different catalytic activity of the 3CuO/α-MnO_2_-*T*-DP catalyst at different calcination temperatures may be related to the dispersion of CuO active sites. In addition, the difference in catalytic activity of 3CuO/α-MnO_2_-*T*-DP catalysts at different calcination temperatures may also be caused by the thermal shrinkage of the catalyst skeleton and the agglomeration of CuO species.

**Figure 10:**
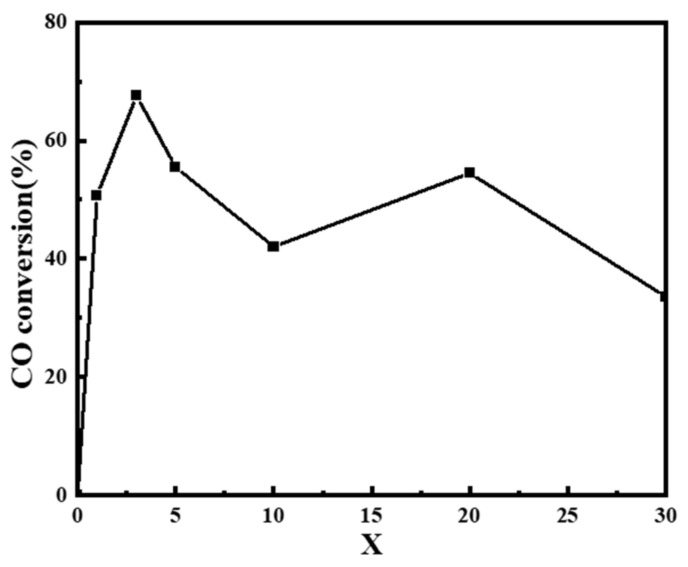
CO conversion and conversion of *x*CuO/α-MnO_2_-200-DP (*x* = 0, 1, 3, 5, 10, 20, 30) catalysts with different CuO loading at 60 °C.

Secondly, the catalytic activity of CO oxidation on the *x*CuO/α-MnO_2_-200-DP catalyst with CuO loading was evaluated in detail, as shown in Figure 9c. As can be seen from the figure, pure α-MnO_2_ nanowire catalyst started to activate at 120 °C. With the increase in reaction temperature, CO conversion gradually increased until reaching 100% at about 200 °C. Compared with pure α-MnO_2_ nanowire carrier, the *x*CuO/α-MnO_2_-200-DP catalyst had higher catalytic activity, especially in the 30–100 °C region. These results indicated that CuO species were the main active center of CO oxidation and the main cause of CO oxidation at a low temperature. To show the difference more clearly in the catalytic activity of the *x*CuO/α-MnO_2_-200-DP catalysts with different CuO loads, the CO conversion of the *x*CuO/α-MnO_2_-200-DP catalysts was analyzed at 60 °C, as shown in Figure 10. When the CuO load increased from 0 wt% to 3 wt%, the catalytic activity of the *x*CuO/α-MnO_2_-200-DP catalyst was significantly enhanced at a low temperature. The 3CuO/α-MnO_2_-200-DP catalyst showed the highest catalytic activity among all the catalysts, which showed a significant advantage compared with previous studies. However, a further increase in CuO from 3 wt% to 30 wt% lead to a decrease in catalytic activity. The possible reason was that the one-dimensional structure of α-MnO_2_ nanowires, especially the aspect ratio of the catalyst, decreases significantly with the increase in CuO load, resulting in a poor dispersion of CuO on the surface of the supports.

Meanwhile, in order to study the influence of the nanowire structure and redox performance of the catalyst support on the catalytic activity, commercial MnO_2_ was used as the contrast carrier for the CO catalytic oxidation reaction and the same loading method was used to prepare the contrast catalyst. Figure 9d shows the catalytic activity of comparative catalysts. In the figure, the 100% CO conversion temperature of the 3CuO/α-MnO_2_-200-DP catalyst was 80 °C, while the CO conversion of the 3CuO/C-MnO_2_-200-DP catalyst was close to 100% only when the temperature was above 200 °C. The catalytic activity of the 3CuO/C-MnO_2_-200-DP catalyst was like the α-MnO_2_ nanowire support without the CuO load. The advantages of the structure and properties of α-MnO_2_ nanowire support were illustrated.

#### 3.2.2. Long-Term Stability Test

**Figure 11:**
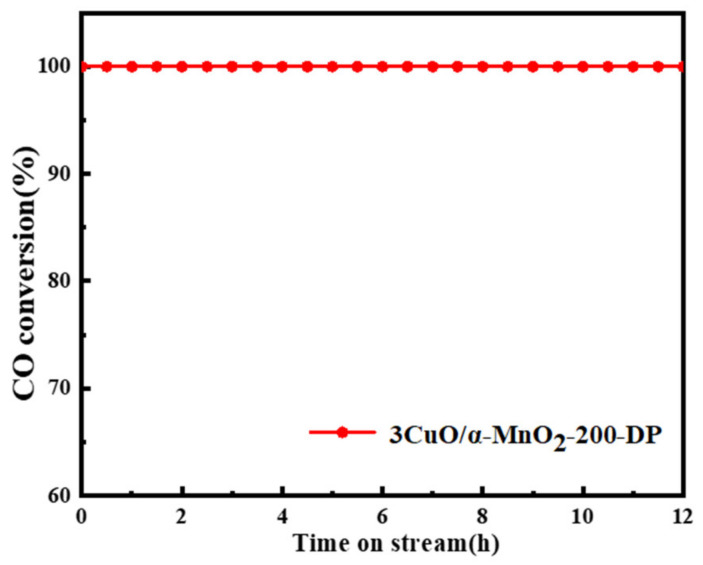
Stability of 3CuO/α-MnO_2_-200-DP catalyst at 80 °C for 12 h.

A long-term stability test was carried out to evaluate the catalytic stability of the prepared CuO-based catalyst under the specific reaction conditions (CO/O_2_/N_2_ = 1/20/79, GHSV = 12,000 mL·g^−1^·H^−1^, 80 °C, 1 atm). The CO oxidation stability of these catalysts was tested for 12 h, and 3CuO/α-MnO_2_-200-DP was selected as the representative catalyst. As can be seen from Figure 11, the 3CuO/α-MnO_2_-200-DP catalyst showed excellent stability during the entire 12 h stability test, with the activity remaining at 100% and no significant deactivation. These results indicate that the 3CuO/α-MnO_2_-200-DP catalyst not only had high temperature activity, but also had good stability.

## 4. Conclusions

In summary, a series of CuO/α-MnO_2_ nanowire catalysts with different CuO loadings, different calcination temperatures and different loading modes were synthesized as the supports of a CO catalytic oxidation catalyst. The slenderness ratio of the CuO/α-MnO_2_ nanowire catalyst decreases with the increase in loading capacity. The results showed that when CuO loading was 3 wt%, calcination temperature was 200 °C and the catalyst that was supported by the deposition precipitation method had the highest catalytic activity. Compared with the commercial MnO_2_ catalyst, the synthetic α-MnO_2_ nanowire catalyst has better redox performance and better low-temperature catalytic activity due to the particularity of the microstructure. Compared with commercial MnO_2_, it was a better catalyst support for CO catalytic oxidation. The CuO particles that were calcined at 200 °C by the precipitation deposition method showed better dispersion on the surface of a α-MnO_2_ nanowire support, which proved that the precipitation deposition method was superior to the traditional initial impregnation method. In addition, CuO as the active center of CO oxidation formed a strong intermetallic synergistic effect on the surface of the α-MnO_2_ nanowire support, which further enhanced the CO oxidation activity of the α-MnO_2_ nanowire support at a low temperature. Because of these advantages, α-MnO_2_ nanowire is considered as a promising support for the CO oxidation of CuO-based catalysts, and has stronger catalytic activity, especially at low temperatures.

## Figures and Tables

**Table 1:** The surface atomic concentration ratio of Mn, Cu, O of the catalysts based on XPS.

Samples	Mn (%)	Cu (%)	O (%)
α-MnO_2_	21.1	/	46.4
1CuO/α-MnO_2_-200-DP	20.8	0.9	45.8
3CuO/α-MnO_2_-200-DP	18.1	2.9	51.1
5CuO/α-MnO_2_-200-DP	18.0	4.0	44.6
10CuO/α-MnO_2_-200-DP	17.8	5.6	43.8
20CuO/α-MnO_2_-200-DP	16.5	7.0	39.4
30CuO/α-MnO_2_-200-DP	10.0	16.9	40.0
3CuO/α-MnO_2_-120-DP	20.4	1.2	45.5
3CuO/α-MnO_2_-300-DP	17.7	2.2	44.4
3CuO/α-MnO_2_-400-DP	16.9	2.0	41.5
3CuO/α-MnO_2_-200-IMP	17.6	1.7	45.9

**Table 2:** O 1 s peak areas of the catalysts based on XPS.

Samples	O 1s Main Peak Area	O 1s Shoulder Peak Area	O 1s Shoulder Peak Area Ratio (%)
α-MnO_2_	129,671.0	32,166.2	19.8
1CuO/α-MnO_2_-200-DP	140,985.7	32,068.2	18.5
3CuO/α-MnO_2_-200-DP	118,838.6	32,724.9	21.6
5CuO/α-MnO_2_-200-DP	138,945.0	32,733.5	19.1
10CuO/α-MnO_2_-200-DP	137,120.8	34,822.0	20.3
20CuO/α-MnO_2_-200-DP	129,100.5	33,846.1	20.7
30CuO/α-MnO_2_-200-DP	129,861.6	32,378.3	20.0
3CuO/α-MnO_2_-120-DP	129,997.5	32,051.5	19.8
3CuO/α-MnO_2_-300-DP	132,452.1	34,305.7	20.6
3CuO/α-MnO_2_-400-DP	134,830.5	33,962.0	20.1
3CuO/α-MnO_2_-200-IMP	134,777.2	32,518.3	19.4

**Table 3:** Binding energies of surface elements in *x*CuO/α-MnO_2_-*T* catalysts.

Samples	Cu 2p_3/2_	O 1s	Mn 2p_3/2_
α-MnO_2_	/	529.8	642.3
1CuO/α-MnO_2_-200-DP	933.2	529.7	642.2
3CuO/α-MnO_2_-200-DP	933.3	529.7	642.2
5CuO/α-MnO_2_-200-DP	933.2	529.6	642.1
10CuO/α-MnO_2_-200-DP	933.2	529.6	642.1
20CuO/α-MnO_2_-200-DP	933.1	529.6	642.1
30CuO/α-MnO_2_-200-DP	933.1	529.6	642.1
3CuO/α-MnO_2_-120-DP	933.2	529.6	642.1
3CuO/α-MnO_2_-300-DP	933.3	529.6	642.1
3CuO/α-MnO_2_-400-DP	933.4	529.7	642.2
3CuO/α-MnO_2_-200-IMP	933.3	529.7	642.2

## Data Availability

The data supporting the findings of this study are available by reason-able request to chenmd@nuist.edu.cn.

## References

[1] Namasivayam A.M., Korakianitis T., Crookes R.J., Bob-Manuel K.D.H., Olsen J. (2010). Biodiesel, Emulsified Biodiesel and Dimethyl Ether as Pilot Fuels for Natural Gas Fuelled Engines. Appl. Energy.

[2] Neidell M.J. (2004). Air Pollution, Health, and Socio-Economic Status: The Effect of Outdoor Air Quality on Childhood Asthma. J. Health Econ..

[3] Prado O.J., Veiga M.C., Kennes C. (2008). Removal of Formaldehyde, Methanol, Dimethylether and Carbon Monoxide from Waste Gases of Synthetic Resin-Producing Industries. Chemosphere.

[4] Lee J.G., An K. (2018). Catalytic Co Oxidation on Nanocatalysts. Top. Catal..

[5] Wang L., Wang L., Zhang J., Wang H., Xiao F.-S. (2018). Enhancement of the Activity and Durability in CO Oxidation over Silica-Supported Au Nanoparticle Catalyst Via Ceox Modification. Chin. J. Catal..

[6] Zheng B., Wu S., Yang X., Jia M., Zhang W., Liu G. (2016). Room Temperature Co Oxidation over Pt/MgFe_2_O_4_: A Stable Inverse Spinel Oxide Support for Preparing Highly Efficient Pt Catalyst. ACS Appl. Mater. Interfaces.

[7] Camposeco R., Hinojosa-Reyes M., Castillo S., Nava N., Zanella R. (2021). Synthesis and Characterization of Highly Dispersed Bimetallic Au-Rh Nanoparticles Supported on Titanate Nanotubes for CO Oxidation Reaction at Low Temperature. Environ. Sci. Pollut. Res..

[8] Zhang X., Li G., Tian R., Feng W., Wen L. (2020). Monolithic Porous CuO/CeO_2_ Nanorod Composites Prepared by Dealloying for CO Catalytic Oxidation. J. Alloys Compd..

[9] Kong F., Zhang H., Chai H., Liu B., Cao Y. (2021). Insight into the Crystal Structures and Surface Property of Manganese Oxide on Co Catalytic Oxidation Performance. Inorg. Chem..

[10] Murthy P.R., Munsif S., Zhang J.-C., Li W.-Z. (2021). Influence of CeO_2_ and ZrO_2_ on the Thermal Stability and Catalytic Activity of Sba-15-Supported Pd Catalysts for Co Oxidation. Ind. Eng. Chem. Res..

[11] Sun L., Zhan W., Li Y.-A., Wang F., Zhang X., Han X. (2018). Understanding the Facet-Dependent Catalytic Performance of Hematite Microcrystals in a Co Oxidation Reaction. Inorg. Chem. Front..

[12] Baidya T., Murayama T., Nellaiappan S., Katiyar N.K., Bera P., Safonova O., Lin M., Priolkar K.R., Kundu S., Rao B.S. (2019). Ultra-Low-Temperature Co Oxidation Activity of Octahedral Site Cobalt Species in CO_3_O_4_ Based Catalysts: Unravelling the Origin of the Unique Catalytic Property. J. Phys. Chem. C.

[13] Lou Y., Wang L., Zhao Z., Zhang Y., Zhang Z., Lu G., Guo Y., Guo Y. (2014). Low-Temperature CO Oxidation over CO_3_O_4_-Based Catalysts: Significant Promoting Effect of Bi_2_O_3_ on CO_3_O_4_ Catalyst. Appl. Catal. B Environ..

[14] Cui Y., Xu L., Chen M., Lv C., Lian X., Wu C.-E., Yang B., Miao Z., Wang F., Hu X. (2019). Co Oxidation over Metal Oxide (La_2_O_3_, Fe_2_O_3_, PrO_2_, Sm_2_O_3_, and MnO_2_) Doped Cuo-Based Catalysts Supported on Mesoporous Ce_0.8_Zr_0.2_O_2_ with Intensified Low-Temperature Activity. Catalysts.

[15] Ren Y., Ma Z., Qian L., Dai S., He H., Bruce P.G. (2009). Ordered Crystalline Mesoporous Oxides as Catalysts for Co Oxidation. Catal. Lett..

[16] Song H., Xu L., Chen M., Cui Y., Wu C.-E., Qiu J., Xu L., Cheng G., Hu X. (2021). Recent Progresses in the Synthesis of MnO_2_ Nanowire and Its Application in Environmental Catalysis. RSC Adv..

[17] Zhao G.-Y., Li H.-L. (2008). Electrochemical Oxidation of Methanol on Pt Nanoparticles Composited MnO_2_ Nanowire Arrayed Electrode. Appl. Surf. Sci..

[18] Ren Y., Ma Z., Dai S. (2014). Nanosize Control on Porous Beta-MnO_2_ and Their Catalytic Activity in Co Oxidation and N_2_O Decomposition. Materials.

[19] Jampaiah D., Velisoju V.K., Venkataswamy P., Coyle V.E., Nafady A., Reddy B.M., Bhargava S.K. (2017). Nanowire Morphology of Mono- and Bidoped Alpha-MnO_2_ Catalysts for Remarkable Enhancement in Soot Oxidation. ACS Appl. Mater. Interfaces.

[20] Du H., Wang Y., Arandiyan H., Younis A., Scott J., Qu B., Wan T., Lin X., Chen J., Chu D. (2017). Design and Synthesis of CeO_2_ Nanowire/MnO_2_ Nanosheet Heterogeneous Structure for Enhanced Catalytic Properties. Mater. Today Commun..

[21] Saputra E., Muhammad S., Sun H., Patel A., Shukla P., Zhu Z.H., Wang S. (2012). Alpha-MnO_2_ Activation of Peroxymonosulfate for Catalytic Phenol Degradation in Aqueous Solutions. Catal. Commun..

[22] Liang S., Bulgan F.T.G., Zong R., Zhu Y. (2008). Effect of Phase Structure of MnO_2_ Nanorod Catalyst on the Activity for Co Oxidation. J. Phys. Chem. C.

[23] Zhang Y., Deng S., Luo M., Pan G., Zeng Y., Lu X., Ai C., Liu Q., Xiong Q., Wang X. (2019). Defect Promoted Capacity and Durability of N-MnO_2-X_ Branch Arrays Via Low-Temperature NH_3_ Treatment for Advanced Aqueous Zinc Ion Batteries. Small.

[24] Selvakumar K., Duraisamy V., Venkateshwaran S., Arumugam N., Almansour A.I., Wang Y., Liu T.X., Kumar S.M.S. (2022). Development of Alpha-MnO_2_ Nanowire with Ni- and (Ni, Co)-Cation Doping as an Efficient Bifunctional Oxygen Evolution and Oxygen Reduction Reaction Catalyst. ChemElectroChem.

[25] Wang J., Luo H., Liu P. (2020). Highly Dispersed Gold Nanoparticles on Metal-Doped Alpha-MnO_2_ Catalysts for Aerobic Selective Oxidation of Ethanol. Catal. Commun..

[26] Li X., Cheng H., Liang G., He L., Lin W., Yu Y., Zhao F. (2015). Effect of Phosphine Doping and the Surface Metal State of Ni on the Catalytic Performance of Ni/Al_2_O_3_ Catalyst. Catalysts.

[27] Hashem A.M., Abuzeid H.M., Narayanan N., Ehrenberg H., Julien C.M. (2011). Synthesis, Structure, Magnetic, Electrical and Electrochemical Properties of Al, Cu and Mg Doped MnO_2_. Mater. Chem. Phys..

[28] Gao J., Jia C., Zhang L., Wang H., Yang Y., Hung S.-F., Hsu Y.-Y., Liu B. (2016). Tuning Chemical Bonding of MnO_2_ through Transition-Metal Doping for Enhanced CO Oxidation. J. Catal..

[29] Zhang Z., Tian Y., Zhao W., Wu P., Zhang J., Zheng L., Ding T., Li X. (2020). Hydroxyl Promoted Preferential and Total Oxidation of Co over Epsilon-MnO_2_ Catalyst. Catal. Today.

[30] Xu R., Wang X., Wang D.S., Zhou K.B., Li Y.D. (2006). Surface Structure Effects in Nanocrystal MnO_2_ and Ag/MnO_2_ Catalytic Oxidation of CO. J. Catal..

[31] Tuan Sang T., Tripathi K.M., Kim B.N., You I.-K., Park B.J., Han Y.H., Kim T. (2017). Three-Dimensionally Assembled Graphene/Alpha-MnO_2_ Nanowire Hybrid Hydrogels for High Performance Supercapacitors. Mater. Res. Bull..

[32] Qian K., Qian Z., Hua Q., Jiang Z., Huang W. (2013). Structure-Activity Relationship of CuO/MnO_2_ Catalysts in CO Oxidation. Appl. Surf. Sci..

[33] Kumar J.P., Ramachatyulu P.V.R.K., Prasad G.K., Singh B. (2015). Montmorillonites Supported with Metal Oxide Nanoparticles for Decontamination of Sulfur Mustard. Appl. Clay Sci..

[34] Papadas I.T., Ioakeimidis A., Vamvasakis I., Eleftheriou P., Armatas G.S., Choulis S.A. (2021). All-Inorganic P-N Heterojunction Solar Cells by Solution Combustion Synthesis Using N-Type FeMnO_3_ Perovskite Photoactive Layer. Front. Chem..

[35] Tafur J.P., Abad J., Roman E., Fernandez Romero A.J. (2015). Charge Storage Mechanism of MnO_2_ Cathodes in Zn/MnO_2_ Batteries Using Ionic Liquid-Based Gel Polymer Electrolytes. Electrochem. Commun..

[36] Kawai J., Maeda K., Nakajima K., Gohshi Y. (1993). Relation between Copper L X-ray Fluorescence and 2p X-ray Photoelectron Spectroscopies. Phys. Rev. B.

[37] Du J., Xiao G., Xi Y., Zhu X., Su F., Kim S.H. (2020). Periodate Activation with Manganese Oxides for Sulfanilamide Degradation. Water Res..

[38] (1932). Mckinney P V, Reduction of palladium oxide by carbon monoxide. J. Am. Chem. Soc..

[39] Freitas I.C., Damyanova S., Oliveira D.C., Marques C.M.P., Bueno J.M.C. (2014). Effect of Cu Content on the Surface and Catalytic Properties of Cu/ZrO_2_ Catalyst for Ethanol Dehydrogenation. J. Mol. Catal. A Chem..

[40] Sun M., Lan B., Lin T., Cheng G., Ye F., Yu L., Cheng X., Zheng X. (2013). Controlled Synthesis of Nanostructured Manganese Oxide: Crystalline Evolution and Catalytic Activities. CrystEngComm.

